# Association between father involvement and attitudes in early child-rearing and depressive symptoms in the pre-adolescent period in a UK birth cohort

**DOI:** 10.1016/j.jad.2017.06.010

**Published:** 2017-10-15

**Authors:** Charles Opondo, Maggie Redshaw, Maria A. Quigley

**Affiliations:** Policy Research Unit in Maternal Health and Care, National Perinatal Epidemiology Unit, Nuffield Department of Population Health, University of Oxford, Old Road Campus, Headington, Oxford OX3 7LF, United Kingdom

**Keywords:** Fathers, Father involvement, Child-rearing, Depression, ALSPAC

## Abstract

**Background:**

Much of the research on parenting and its influence on child development has emphasised the mother's role. However, increasing evidence highlights the important role of fathers in the development, health and well-being of their children. We sought to explore the association between paternal involvement in early child-rearing and depressive symptoms in 9 and 11 year-old children.

**Methods:**

We used data from the Avon Longitudinal Study of Parents and Children (ALSPAC) cohort recruited in the southwest of England. The outcome was depressive symptoms measured using the short Moods and Feelings Questionnaire (sMFQ) score. The main exposure was father involvement measured through factor analysis of fathers’ responses on their participation in, understanding of, and feelings about their child's early upbringing. Scores on factor 1 measured fathers’ emotional response to the child; scores on factor 2 measured the frequency of father involvement in domestic and childcare activities; scores on factor 3 measured fathers’ feelings of security in their role as parent and partner.

**Results:**

Children of fathers with high scores on factors 1 and 3 had 13% (OR 0.87, 95%CI 0.77–0.98, p = 0.024) and 9% (OR 0.91, 95%CI 0.80–1.03, p = 0.129) respectively lower adjusted odds of depressive symptoms at 9 and 11 years. For factor 2, there was weak evidence of a 17% increase in odds of depressive symptoms associated with 1 unit higher factor scores at both ages (OR 1.17, 95%CI 1.00–1.37, p = 0.050).

**Limitations:**

In these observational data, the possibility of residual confounding in the association between the exposure and the outcome cannot be ruled out.

**Conclusion:**

Positive psychological and emotional aspects of father involvement in children's early upbringing, but not the quantity of direct involvement in childcare, may protect children against developing symptoms of depression in their pre-teen years.

## Highlights

• Emotionally responsive parenting by fathers is associated with less depression in pre-teenagers.
• Paternal involvement in child-rearing appears to be associated with more depression.
• Adjusting for paternal work hours and socioeconomic status attenuates the latter association.

## 1 Introduction

Depression is an increasingly common cause of morbidity and mortality globally (Murray and Lopez, 1996) affecting adults and teenagers in almost equal measure in some populations (Kessler et al., 2005). About 2% of children may already be affected by depression by the time they reach their pre-teen and early teenage years, and this may put them at risk of later mental health problems (Copeland et al., 2013). The aetiology of childhood depression is complex, and a variety of genetic and environmental factors are thought to contribute to this outcome (Rice et al., 2002; Scourfield et al., 2003). Increasing concern about the mental health of young people has led to considerable effort directed at supporting the evidence base (Kieling et al., 2011; Patel et al., 2007) and improving the environment in which children are raised to facilitate better outcomes (Glass, 1999; Olds, 2006). The nature of early parenting plays a central role in creating a favourable or unfavourable family environment which have the potential to foster better or poorer outcomes for children (Desforges and Abouchaar, 2003; Raby et al., 2015; Schneider et al., 2001).

The effect of paternal involvement in a child's early upbringing on later mental health outcomes is poorly understood. Diagnosis and measurement of later depressive symptoms has often been included in studies of the impact of family conflict, separation and divorce and parental pathology, for example, maternal and paternal depression (Chang et al., 2007; Pettit et al., 2001), with the emphasis on negative outcomes for children (Sandler et al., 2008, Simons et al., 1999). Father absence in early childhood and potential child abuse at the toddler stage have both been associated with depressive symptoms in the middle teens (Culpin et al., 2013; Scourfield et al., 2016).

However, an increasing body of evidence shows that father involvement in child-rearing can positively influence a variety of child developmental outcomes. Children with more involved fathers have been observed to exhibit fewer behavioural problems (Amato and Rivera, 1999; Carlson, 2006; Dex and Ward, 2007), have a lower tendency to engage in risky behaviour such as teenage smoking (Menning, 2006), delinquency (Carlson, 2006) and contact with law enforcement (Flouri and Buchanan, 2002a). They also have better cognitive (Nugent, 1991) and educational outcomes (Flouri and Buchanan, 2004) and experience better peer (Pruett, 1997) and partner (Flouri and Buchanan, 2002b) relationships, among a range of other positive effects (Allen and Daly, 2007). Yet a number of studies have also failed to demonstrate the positive effects of father involvement on child outcomes (Aldous and Mulligan, 2002; Cabrera and Tamis-LeMonda, 2000; Huerta et al., 2013; Sullivan et al., 2010).

Most studies of father involvement conceptualise it as a unidimensional construct (Brunton et al., 2010). For example, in a review of longitudinal studies on the effects of father involvement on various child outcomes (Allen and Daly, 2007) most studies were found to measure father involvement by the amount of father-child interaction, direct engagement in childcare, being a resident father or frequency of visitation. However, father involvement is a complex construct, comprising interaction, care and attitudes to parenting in addition to financial provision. Societal views of the role of fathers in child-rearing have changed over time along with views and policies relating to family life. Fathers have been regarded as patriarchs, moral teachers, gender role models, providers and, more recently, as active nurturers (Lamb, 2010). Thus the mixed evidence on the positive effects of paternal involvement in child development and longer term outcomes may be partly attributable to the different ways the construct has been measured. It also may be affected by the time-points chosen for investigation, with more proximal outcomes commonly selected. The biological, cognitive and social changes associated with puberty make pre and early adolescence a critical transition period and of interest in gaining an understanding of the possible impact of early parenting (Lamb and Lewis, 2010).

Recent research has acknowledged the multidimensional nature of father involvement. We have previously reported an approach to measuring father involvement based on factor analysis which identified specific aspects, namely engagement in domestic and childcare activities, emotional response to the baby and parenting, and security in the role of parent and partner (Opondo et al., 2016). This approach, which utilised data from a longitudinal cohort, examined the role of fathers beyond their mere presence in the home and financial provision. It recognises the complex nature of father engagement and allows for independent exploration of the effects of the different aspects of involvement on child outcomes. In the current study we extended this approach to explore whether different aspects of involvement of resident fathers were associated with depressive symptoms in pre-adolescent children. We hypothesised that children who benefitted from greater paternal involvement in their very early years would be less likely to exhibit symptoms of depression in their pre-teen years.

## 2 Methods

**Fig. 1:**
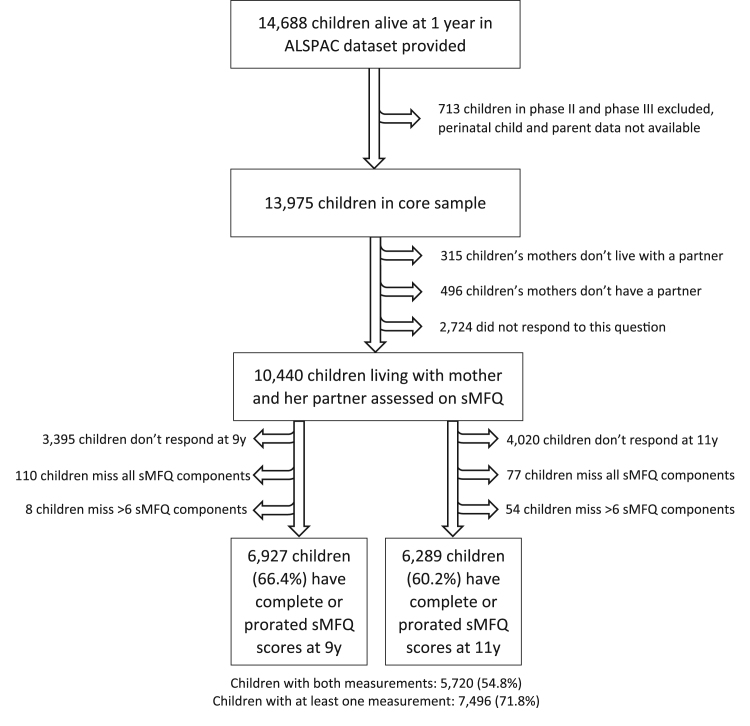
Sample profile of the children included in the analysis.

This was an observational study for which the data were drawn from the Avon Longitudinal Study of Parents and Children (ALSPAC). The ALSPAC study is a cohort of children born in the southwest of England between April 1991 and December 1992. The study design, methodology and cohort profile have been described elsewhere in detail (Boyd et al., 2012; Fraser et al., 2013; Golding, 1990; Golding et al., 2001) and the study website contains all the data and a fully searchable data dictionary (ALSPAC Study Team, 2016). Briefly, the cohort included 14,701 children in the total sample who were alive at 1 year. Observations on 13 triplet or higher-order multiple births were omitted to preserve confidentiality. Another 713 children who were not in the initial ‘phase I’ sample were excluded, as were 3535 children who were not reported to be living with their father or a male partner of their mother in the first year. Thus 10,440 children were eligible for our study. Of these, we have complete or prorated scores of the outcome measure for 6927 children at 9 years and 6289 at 11 years, with 5720 children having both measurements and 7496 having at least one measurement (Fig. 1).

Data included in this analysis were collected using self-completion questionnaires sent to families after recruitment and when the child was aged 8 weeks, 8 months, 9 years and 11 years. The questionnaires asked about mental health, parenting and childcare, behaviour, socio-economic status of parents, and child development.

The outcome was depressive symptoms in the child measured by the short Moods and Feelings Questionnaire (sMFQ) completed by the child (Messer et al., 1995) at 9 and 11 years. The sMFQ is a unidimensional self-report tool whose items have good discrimination at the severe end of the latent trait, and have been shown to have good internal construct validity and adequacy for measuring depressive symptoms (Messer et al., 1995; Sharp et al., 2006). It has been validated for use in identifying signs and symptoms of depression in children and adolescents. The measure has 13 negatively-worded statements about one's mood and feelings, each eliciting one of three ordered responses: 0 (‘not true’), 1 (‘sometimes) or 2 (‘true’). The item scores are summed up to obtain a total score.

The main exposure was father involvement which was measured with factor scores derived from the factor analysis of 45 items rated by fathers. These items were designed by the ALSPAC team and not drawn from an existing measure. Fathers were asked to rate their level of agreement, on 3–6 point ordinal response scales, with statements reflecting their involvement with direct care and associated household tasks, attitudes to parenting, relationship with their child, and moods and feelings, 8 weeks and 8 months after the birth of the child. Results of the factor analysis are available elsewhere (Opondo et al., 2016). Three domains of father involvement were identified in the factor analysis, namely ‘emotional response to baby and parenting’ (Factor 1), ‘engagement in domestic and childcare activities’ (Factor 2), and ‘security in role as parent and partner’ (Factor 3).

Potential moderators of the association between the outcome and exposure variables were identified from maternal and paternal responses to the questionnaires at 8 weeks and 8 months postnatally. These included parents’ age, level of education (O-level/CSE/vocational training, A-level or university degree), depressive symptoms measured 8 weeks postnatally on the Edinburgh Postnatal Depression Scale (EPDS) (Murray and Carothers, 1990), socio-economic status derived from self-reported occupation using the Computer Assisted Structured Coding Tool (CASCOT) and coded into quintiles from 1 (lowest) to 5 (highest) (Warwick Institute for Employment Research, 2000), parity, number of hours worked in the current or most recently held job, return to work by mother, and child's age and gender. Given that level of education and occupation-based socio-economic status were likely to be highly correlated within and between parents, we derived a family socio-economic status variable by conducting a principal component analysis of both parents’ SES and education level variables, extracting the first principal component and recoding it into three equal-sized categories from 1 (lowest) to 3 (highest).

We performed a longitudinal analysis in which the outcomes measured at 9 years and 11 years were fitted in the same model. Association between father involvement and child depression was explored by fitting hierarchical ordinal logistic regression models of the two sMFQ scores measured in each child. First, univariable models of sMFQ scores were fitted for each explanatory variable. Linear trends were explored in ordered categorical explanatory variables and those recoded from continuous variables when necessary (e.g. variables with few observations for some values across the expected range). Next, explanatory variables associated with sMFQ in the univariable models were included in separate multivariable models for each of the three domains of father involvement, retaining child's age, child's gender and any explanatory variables that remained associated with the outcome in the adjusted models at both time-points based on p-values of 5% or less. Family socioeconomic status (SES) was also retained in the final models irrespective of p-value since an imbalance in the prevalence of depression has previously been observed across various SES levels (Elovainio et al., 2012; Gilman et al., 2002; Ochi et al., 2014). We investigated a difference in association between the main exposures and outcome in boys versus girls and across the two time-points by testing for interaction by sex and time respectively. Proportionality of odds in the ordinal logistic regression models was tested using the Brant test (Long and Freese, 2006) on parallel regression models of the outcome at 9 and 11 years, and listwise deletion was used to deal with missing data. Data management and manipulation were performed in Stata v13 (StataCorp LP, 2014).

## 3 Results

**Fig. A1:**
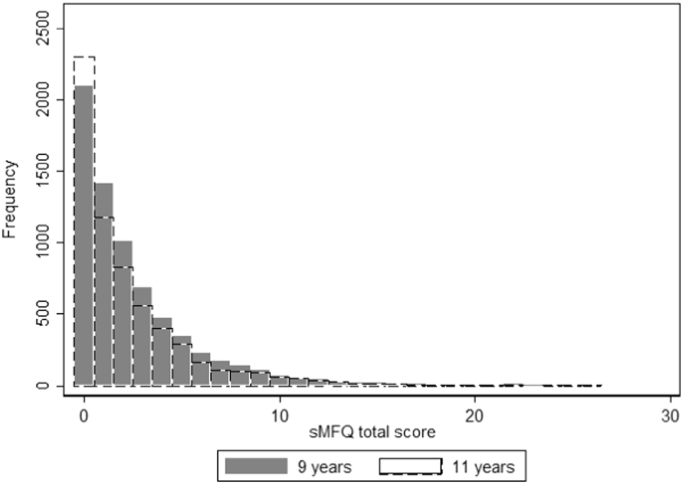
Distribution of total sMFQ scores at ages 9 and 11 years.

The analysis was based on the 7496 children with at least one sMFQ measurement at age 9 or 11 (Table 1). There were slightly more boys than girls in this sample. Most parents were educated to O-level, CSE (awarded on completing compulsory schooling at age 16 in England) or had some vocational training. The distributions of socioeconomic status categories in fathers and mothers were similar, with most falling in the middle category. Fathers were on average 2 years older than mothers (mean age 31.3 vs. 29.3 years) and had lower EPDS depression scores (median 3 vs. 5). Fathers reported working about 45 h per week in their current or most recent job around the time when the child was 8 months old and most mothers had not returned to work (or their usual occupation) by the time their child was 8 weeks (93.3%) or 8 months (63.2%) old. On average mothers had 1 child prior to the one included in the study. Children had low sMFQ scores overall, with a median score of 1. The distribution of sMFQ scores is shown in the Appendix in Fig. A1. Only 3.5% of children had sMFQ scores of 12 or more on at least one measurement time-point, which may signify depression (Messer et al., 1995).

**Table 1:** Characteristics of the parents and children included in the analysis.

	**n = 7496**
**Fathers**	
Age in years 18 weeks after birth of child, mean (SD)	31.18 (5.4)
Highest level of education, n (%)	
O-level, CSE^a^ or vocational	3325 (44.4%)
A-level^b^	1563 (20.9%)
University degree	1092 (14.6%)
Missing	1516 (19.0%)
Hours worked per week, mean (SD)	44.88 (9.9)
EPDS^c^ score 8 weeks after birth of child, median (IQR)	3 (1–6)
SES^d^ category, n (%)	
1 – lowest	156 (2.1%)
2	555 (7.4%)
3	2758 (36.8%)
4	2524 (33.7%)
5 – highest	916 (12.2%)
Missing	587 (7.8%)
**Mothers**	
Age in years at birth of child, mean (SD)	29.2 (4.4)
Highest level of education, n (%)	
O-level, CSE or vocational	2822 (37.7%)
A-level	1792 (23.9%)
University degree	1472 (19.6%)
Missing	1410 (18.8%)
Parity, median (IQR)	1 (0–1)
Return to work or school 8 weeks after birth of child, n (%)	
Yes	505 (6.7%)
No	6991 (93.3%)
Return to work or school 8 months after birth of child, n (%)	
Yes	2752 (36.7%)
No	4734 (63.2%)
Missing	10 (0.1%)
EPDS score 8 weeks after birth of child, median (IQR)	5 (2–8)
SES^d^ category, n (%)	
1 – lowest	104 (1.4%)
2	498 (6.6%)
3	3092 (41.3%)
4	2.230 (29.8%)
5 – highest	460 (6.1%)
Missing	1112 (14.8%)
**Children**	
Mean age difference in months, mean (SD)	0.00 (5.8)
Gender, n (%)	
Boys	3705 (50.6%)
Girls	3791 (49.4%)
sMFQ^e^ score, median (IQR)	1 (0–3)
sMFQ score of 12 or more on at least 1 measurement, n (%)	259 (3.5%)

a O-level and CSE were the national exams which students in England sat in their last year of compulsory school education at age 16.

b A-levels are pre-university examinations.

c EPDS is the Edinburgh Postnatal Depression Scale.

d SES is socioeconomic status based on individual's occupation, derived from the Computer-assisted structured coding tool (CASCOT).

e sMFQ is the short Mood and Feelings Questionnaire.

In the unadjusted ordered logistic regression there was evidence that all three domains of paternal involvement were associated with the outcome. A positive emotional response to the baby and parenting (Factor 1), and greater paternal security in the parent and partner role (Factor 3) were associated with 33% and 39% reduction in proportional odds of higher sMFQ scores, respectively. Higher levels of paternal engagement in domestic and child care tasks (Factor 2) were, however, associated with 21% *increased* proportional odds of higher sMFQ scores in children (Table 2).

**Table 2:** Crude and adjusted proportional odds ratios for the effect of paternal involvement on sMFQ scores, with 95% confidence intervals and p-values.

Paternal involvement factor scores	**Crude**^a^ **[n = 6157]**			**Adjusted**^b^ **[n = 5191]**		
	**Odds ratio**^c^	**95% confidence interval**	**p-value**	**Odds ratio**^c^	**95% confidence interval**	**p-value**
Factor 1: “emotional response to baby and parenting”	0.67	0.60–0.75	< 0.001	0.87	0.77–0.98	0.024
Factor 2: “engagement in domestic and childcare activities”	1.21	1.03–1.41	0.019	1.17	1.00–1.37	0.050
Factor 3: “security in role as parent and partner”	0.61	0.55–0.69	< 0.001	0.91	0.80–1.03	0.129

a Adjusted for time of outcome measurement.

b Adjusted for time of outcome measurement, paternal and maternal depression, parity, child's age and gender, and family socioeconomic status.

c Proportional odds ratio for higher sMFQ scores.

Other factors were found to be associated with the outcome (Table 3). Both paternal and maternal depression at 8 weeks were associated with increased proportional odds of higher sMFQ scores, as was child's age. Increased parental age and higher parity were associated with reduced proportional odds of higher sMFQ scores. All these factors were included in the adjusted model, along with child's gender and family socioeconomic status as *a priori* potential confounders. Parental age was not associated with the outcome in the adjusted models and was therefore subsequently excluded from further analyses.

**Table 3:** Univariate proportional odds ratios for the associations between paternal, maternal and child characteristic with sMFQ scores at ages 9 and 11 years.

	Odds ratio^a^	95% confidence interval	p-value
Fathers			
Age at birth of child, years	0.99	0.98 – 1.00	0.077
Highest level of education	1.01	0.94 – 1.10	0.740
Hours worked per week	1.03	0.95 – 1.12	0.505
Depression, EPDS	1.09	1.07 – 1.11	<0.001
SES^b^ category	0.99	0.93 – 1.06	0.867
Mothers			
Age at birth of child, years	0.98	0.97 – 0.99	0.004
Highest level of education	0.97	0.90 – 1.05	0.483
Parity	0.92	0.86 – 0.98	0.009
Return to work or school at 8 weeks	1.03	0.83 – 1.28	0.806
Return to work or school at 8 months	1.10	0.98 – 1.23	0.110
Depression, EPDS	1.15	1.13 – 1.16	<0.001
SES^b^ category	0.98	0.91 – 1.06	0.607
Children			
Age, months	1.02	1.01 – 1.03	0.001
Gender, boys	0.95	0.85 – 1.06	0.368
Family			
SES^c^ category	1.01	0.94 – 1.08	0.833

a proportional odds ratio for higher sMFQ scores.

b Socioeconomic status based on individual's occupation, derived from the Computer-assisted structured coding tool (CASCOT).

c Family socioeconomic status derived from principal components analysis of fathers’ and mothers’ SES and level of education.

**Fig. 2:**
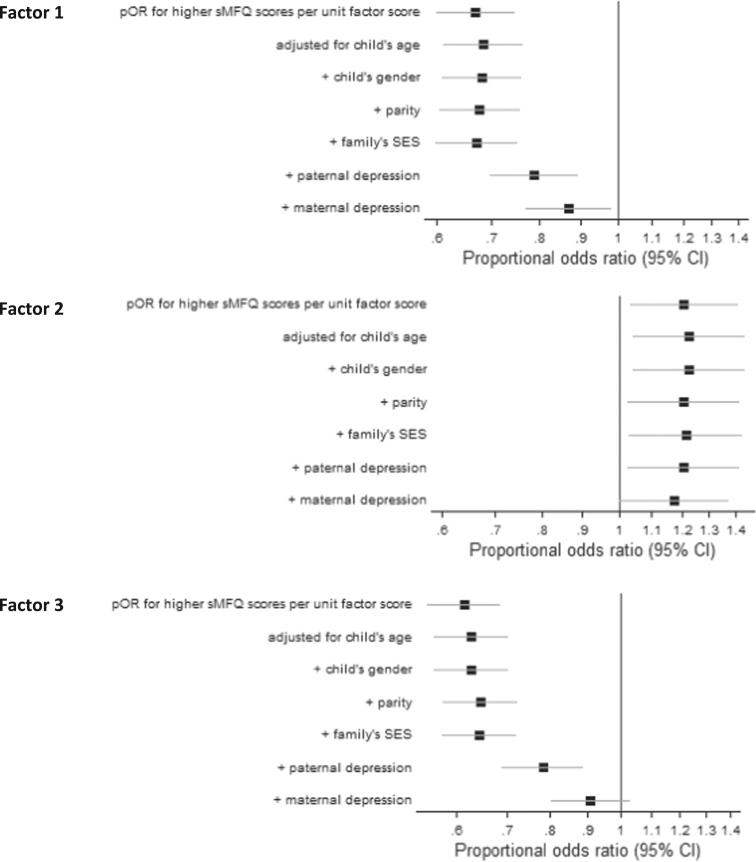
Systematic adjustment of the association between father involvement factor scores and sMFQ scores measured at 9 and 11 years.

There was strong evidence of a 13% reduction in proportional odds of higher sMFQ scores in the adjusted model for paternal involvement on Factor 1 comparing children of the same age, gender, parental depression, parity and family socioeconomic status (Table 2). Factor 2 was associated with a 17% adjusted increase in proportional odds of higher sMFQ scores, although the evidence for association was weak. For Factor 3, there was no evidence of association for the 9% reduction in odds of higher sMFQ scores. Parental depression was most responsible for the attenuated magnitude of association between the unadjusted and adjusted models (Fig. 2). No evidence of a difference in association for boys versus girls or for a difference in effect of paternal involvement across the two time-points was observed in the tests for interaction. In each of these models the proportional odds assumption was not violated; Brant test p-values in adjusted non-imputed models of the effect of the three respective domains of paternal involvement conducted in parallel for the two time-points were 0.339, 0.186 and 0.299 at 9 years and 0.940, 0.947 and 0.964 at 11 years.

## 4 Discussion

We have described a secondary analysis of data from a birth cohort to explore the association between father involvement early in their children's lives with later depressive symptoms in the children. Our analysis provides evidence that when fathers expressed emotionally responsive attitudes to their baby early in their child's life, their children tended to report fewer symptoms of depression in their pre-teen years. However, there was no evidence of association between paternal security in their parent/partner role and reported symptoms of depression in pre-teens. We also found weak evidence that greater father engagement in domestic and childcare activities was associated with an increase in the odds of reporting more symptoms of depression by pre-teenage children.

Our findings are consistent with previous studies which suggest that paternal warmth (Alloy et al., 2001), positive direct paternal involvement with their child (Sanford et al., 1995) and the quality of father-partner relationships (Downey and Coyne, 1990) during a child's adolescence may predict depression. Our findings further suggest that these associations may also exist between father involvement early in a child's life and later child outcomes up to the pre-adolescent period. This is supported by a smaller prospective study linking minimal father involvement at 12 months with poor mental health outcomes at nine years (Boyce et al., 2006). Associations of the kind described by Flouri and others on the role of paternal involvement in childhood and the teenage years and adult outcomes, including mental health, reflect both continuities in parenting and the benefits of early and later involvement (Flouri, 2005; Flouri and Buchanan, 2002c).

There was weak evidence that children of fathers who were more involved in domestic and childcare activities were more likely to report symptoms of depression in the pre-teen years. This finding contradicts established evidence which shows either a positive (Allen and Daly, 2007; Boyce et al., 2006) or null association (Kroll et al., 2016) between this form of paternal involvement and child mental health outcomes. Indeed this finding may be one manifestation of the many complex ways in which family circumstances can influence both the nature of parental involvement and child outcomes, with unexpected consequences. We observed that fathers who worked fewer hours every week – who also tended to be less well-off according to the CASCOT social classification – were more likely to report greater involvement in domestic and childcare activities. It is well documented that fathers in full-time employment are comparatively less active in childcare (Aldous et al., 1998), and for unemployed fathers or those working part-time, providing childcare may be a necessity rather than a choice (Allen and Daly, 2007). Thus the observed association between paternal involvement and pre-teen depression may be the confluence of greater involvement in domestic and childcare activities among underemployed and less well-off fathers, and prevalent depression among socially disadvantaged pre-teens (Elovainio et al., 2012, Gilman et al., 2002). This interpretation is supported by the finding that further adjusting our models for paternal work hours attenuated the negative association of father involvement in domestic and childcare activities with pre-adolescent depression in children.

There are a number of possible mechanisms by which early father involvement may affect pre-adolescent depression. A warm father-child relationship may foster a healthy attachment which may then protect the child against developing depressive symptoms. Although evidence for the effect of father-child attachment on child depression is limited, there is evidence that mother-child attachment may influence child depression (Woodward et al., 2000). Paternal involvement may also be integral to a harmonious inter-partner relationship, and there is evidence to support the association between the quality of the partner relationship and child adjustment (Gable et al., 1994).

We measured father involvement by calculating factor scores from items representing fathers’ own report of their involvement in direct child-care, household tasks, attitudes to parenting, relationship with child and partner and their moods and feelings in the first year after the birth of the child (Opondo et al., 2016). The underlying factors were broadly consistent with Pleck's conceptualisation of father involvement (Pleck, 2010) which classified positive engagement activities, warmth and responsiveness, and control as ‘primary’ components and indirect care and process responsibility as ‘auxiliary’ components of father involvement. However, measuring father involvement solely from fathers’ own perspective was a potential source of bias which may be reduced by including elements of maternal report of father involvement in the measurement (Mizell, 2002).

Estimates of the association between father involvement and child depression were adjusted for parental depression, parity, family socioeconomic status, and child's age and gender, each of which was either already known to be associated with the outcome or observed to be associated in these data. For example, it is well established that depressive disorders become more prevalent in girls during adolescence (Angold et al., 1996; Hankin et al., 1998; Nolen-Hoeksema and Girgus, 1994) hence the need to adjust for the potential effect of age and gender. There's also very strong evidence for a deleterious effect of parental depression, especially in a child's early years, on a variety of mental health outcomes including depression (Cox, 2005; Cummings and Davies, 1994; Murray, 2004; Schumacher et al., 2008) especially for girls (Nilsen et al., 2013). In each case, the adjustment appeared to attenuate the magnitude and strength of evidence of the observed association between father involvement and child depression. The possibility of residual confounding by some other unmeasured factors which could potentially further attenuate the associations that we have observed cannot be ruled out.

Other limitations to this study include limitations on the generalisability of findings from a cohort born over 25 years ago to the present day and the attrition of the original sample through non-response and losses to follow-up which could precipitate selection bias.

Future research could consider a wider range of possible moderating variables, for example, infant temperament and intervening adversity on mental health in pre-adolescence, for which links with later mental health have been reported (Bould et al., 2014; Scourfield et al., 2016). More broadly it would be useful to look more at the impact of fathers, their relationships with their children and outcomes in other situations, including low and middle income country contexts (Verkuijl et al., 2014).

### 4.1 Clinical and policy implications

Mental health, particularly depression in young people is acknowledged to be of considerable concern. There have been moves to prioritise ensuring a good start in life, early intervention across all ages, with an emphasis on taking a life course approach, with the objective of improving outcomes at all ages in the UK (Department of Health, 2011; The Mental Health Taskforce, 2016) and elsewhere (Lawrence et al., 2015; Mental Health Commission of Canada, 2012).

Poor parental health is known to impact negatively on outcomes for children and young people, however, protective factors and those associated with vulnerability and risk may not work in a converse manner and interventions must take account of this (Rutter, 1989; Rutter and Rutter, 1993). The low levels of depressive symptoms evidenced in the pre-adolescent period, in families where both parents were resident early on, are as might be anticipated. However, where paternal involvement has been absent or extreme the negative associations referred to persist into the teenage years (Culpin et al., 2015, Culpin et al., 2013; Scourfield et al., 2016) and later (McLanahan et al., 2013) and while family oriented interventions may affect exposure, later interventions will need to focus on individual identification, support and treatment.

## 5 Conclusion

The findings of this study show that positive psychological and emotional involvement by fathers in the upbringing of their children in infancy, but not the amount of time engaged in childcare and domestic tasks, may protect children for developing symptoms of depression later in life. This protective effect may persist up to the pre-teen years, but not necessarily into the early teen years when other factors may become more powerful determinants of mental health status.

## Role of the funding source

This paper reports on an independent study which was funded by the Policy Research Programme in the Department of Health, England (grant 108/0001). The views expressed are not necessarily those of the Department. The UK Medical Research Council (Grant ref: 102215/2/13/2) and the Wellcome Trust (grant 102215/2/13/2), and the University of Bristol provide core support for ALSPAC.

The funders had no role in the design, conduct, analyses or writing of this study or in the decision to submit for publication.

## Appendix

See Fig. A1 here
